# Gastroparesis and lipid metabolism-associated dysbiosis in Wistar-Kyoto rats

**DOI:** 10.1152/ajpgi.00008.2017

**Published:** 2017-04-13

**Authors:** J. E. Dalziel, Karl Fraser, Wayne Young, Catherine M. McKenzie, Shalome A. Bassett, Nicole C. Roy

**Affiliations:** ^1^Food Nutrition and Health Team, Food and Bio-Based Products Group, AgResearch Grasslands Research Centre, Palmerston North, New Zealand;; ^2^Bioinformatics Mathematics and Statistics, AgResearch, Palmerston North, New Zealand; and; ^3^Riddet Institute, Massey University, Palmerston North, New Zealand

**Keywords:** gastrointestinal transit, irritable bowel syndrome, anxiety, bile acid, microbiota

## Abstract

Altered gastric accommodation and intestinal morphology suggest impaired gastrointestinal (GI) transit may occur in the Wistar-Kyoto (WKY) rat strain, as common in stress-associated functional GI disorders. Because changes in GI transit can alter microbiota composition, we investigated whether these are altered in WKY rats compared with the resilient Sprague-Dawley (SD) rats under basal conditions and characterized plasma lipid and metabolite differences. Bead transit was tracked by X-ray imaging to monitor gastric emptying (4 h), small intestine (SI) transit (9 h), and large intestine transit (12 h). Plasma extracts were analyzed by lipid and hydrophilic interaction liquid chromatography (HILIC) and liquid chromatography-mass spectrometry (LC-MS). Cecal microbial composition was determined by Illumina MiSeq 16S rRNA amplicon sequencing and analysis using the QIIME pipeline. Stomach retention of beads was 77% for WKY compared with 35% for SD rats. GI transit was decreased by 34% (9 h) and 21% (12 h) in WKY compared with SD rats. Excluding stomach retention, transiting beads moved 29% further along the SI over 4–9 h for WKY compared with SD rats. Cecal *Ruminococcus*, *Roseburia*, and unclassified *Lachnospiraceae* genera were less abundant in WKY rats, whereas the minor taxa *Dorea*, *Turicibacter*, and *Lactobacillus* were higher. Diglycerides, triglycerides, phosphatidyl-ethanolamines, and phosphatidylserine were lower in WKY rats, whereas cholesterol esters and taurocholic acids were higher. The unexpected WKY rat phenotype of delayed gastric emptying, yet rapid SI transit, was associated with altered lipid and metabolite profiles. The delayed gastric emptying of the WKY phenotype suggests this rat strain may be useful as a model for gastroparesis.

**NEW & NOTEWORTHY** This study reveals that the stress-prone Wistar-Kyoto rat strain has a baseline physiology of gastroparesis and rapid small intestine transit, together with metabolic changes consistent with lipid metabolism-associated dysbiosis, compared with nonstress-prone rats. This suggests that the Wistar-Kyoto rat strain may be an appropriate animal model for gastroparesis.

View this article's corresponding video summary at https://youtu.be/BeI39Jh2BLk.

## Abstract (precis)

This study reveals that the stress-prone Wistar-Kyoto rat strain has a baseline physiology of gastroparesis and rapid small intestine transit, together with metabolic changes consistent with lipid metabolism-associated dysbiosis, compared with nonstress-prone rats. This suggests that the Wistar-Kyoto rat strain may be an appropriate animal model for gastroparesis.

animal models of anxiety and depression are often used to study functional gastrointestinal (GI) disorders because these can be considered disorders of the gut-brain axis. The Wistar-Kyoto (WKY) rat strain is sensitive to stress and is used as a model for anxiety (24), depression (36) and visceral hypersensitivity (19), yet little is known about its GI function and microbiota compared with the Sprague-Dawley (SD) strain, which is often used as a resilient comparison (21, 32, 33). WKY rats are reported to have impaired gastric accommodation such that their maximal volume response to stomach distension is decreased compared with SD rats (31a), but peristaltic motility does not differ (23). However, whether this impairment in the WKY strain affects gastric emptying or GI transit during digestion in other regions is not known. WKY rats exhibit altered colonic morphology associated with mild mucosal damage (34) and show stress-induced changes in intestinal transport (44, 45). They also show significantly higher anxiety-induced defecation during an open field test than SD rats (34). While this suggests that GI transit may also be altered, as occurs in intestinal stress-associated functional disorders, fecal pellet output does not always correlate with colonic transit in response to stress (31).

Understanding the relationship between stress/anxiety and GI transit may help develop long-term approaches to self-management of mild dysmotility, for example, through dietary intervention. Defining the phenotype of the stress-prone WKY strain and determining functional differences compared with the SD strain will better enable use of this strain as a model for human functional GI disorders and may enable identification of associated biomarkers.

To investigate whether strain differences in GI function exist, we compared GI transit and microbiota composition between WKY and SD rats. Furthermore, other studies have shown that changes in GI transit can be associated with altered microbiota profiles (49) and may be reflected in circulating metabolite profiles (41). We therefore investigated possible differences in plasma metabolite and lipid concentrations between WKY and SD strains. We used an established high-resolution imaging method to track GI transit of solid matter from the stomach to the distal colon in vivo (11).

To achieve high-resolution images, we used an X-ray imaging system, which can readily determine relative rates of transit between regions. We measured different regions by tracking the transit of six metallic beads over 12 h to assess difference in stomach emptying and in small and large intestinal transit. Nonfasted rats were used to maintain normal digestion and transit and to avoid retention of beads in the stomach as has been reported for solid capsules in fasted rats (43). The beads were given with a barium slurry providing a mix of solid and semisolid gastric contents, which approximates human measurement techniques (1). Differences in the intestinal microbiota between rat strains were examined and compared with corresponding changes in microbiota reported for irritable bowel syndrome. We utilized nontargeted liquid chromatography-mass spectrometry (LC-MS)-based metabolomics to detect differences between both rat strains by classifying into them two groups and identifying key compounds that contribute to this difference.

## MATERIALS AND METHODS

### Animal Care

This study was conducted with ethical approval (application AE13501) by the AgResearch Grasslands Animal Ethics Committee (Palmerston North, New Zealand) in accordance with the Animal Welfare Act, 1999 (New Zealand). Male SD and WKY rats (12 of each strain) were purchased from the Animal Resources Centre (Canning Vale, WA, Australia). The animals were raised in groups then housed individually 7 days before commencement of the study (9 wk of age) at a constant temperature of 21°C and maintained under a 12-h:12-h light-dark cycle, lights on 08:00. At 10 wk of age they were fed an AIN-93M diet (OpenStandard Rodent Diet; Research Diets. New Brunswick, NJ) and water, provided ad libitum for 2 wk. The animals were monitored three times weekly for weight, food intake, and General Health Score (1–5; New Zealand Animal Health Care Standard). At the end of the study the rats were euthanized using carbon dioxide inhalation overdose.

### GI Transit Procedures and Measurements

The methods used have been described previously (11, 43). Each rat received six solid, stainless steel beads, diameter (*d*) = 1.4 mm (Bal-tec, Los Angeles, CA) via oral gavage in 2 ml of 15% barium sulfate in slurry form (E-Z-HD 98% wt/wt; Cat. No. 764; E-Z-EM Canada, kindly provided by Palmerston North Hospital, New Zealand). Note that for the bead size comparison experiments smaller beads (*d* = 1.1 mm) were also used. Isoflurane anesthesia was induced in a chamber and persisted for 5 min during which gavage was performed upon recovery of the swallow reflex.

#### X-ray imaging.

GI transit was tracked at three time points by X-ray imaging while rats were under brief 5% isoflurane anesthesia to monitor: exit from stomach (4 h), small intestinal transit (9 h), and large intestinal transit (12 h). The metallic beads were visualized by X-ray, and the relatively opaque barium sulfate outlined the GI tract, enabling identification of bead location.

Ventral and right lateral views were taken using a portable X-ray unit (Porta 100HF 2.0kW High Frequency; Job, Yokohama, Japan) including a camera and digital cassette (Canon 55G DR sensor panel) in conjunction with a laptop computer (Lenovo ThinkPad W530) and image-viewing software (Lenovo ThinkPad W530). Image files (DICOM) were visualized using MicroDicom DICOM Viewer v8.7 (Simeon Antonov Stoykov, Sofia, Bulgaria).

#### Stomach emptying.

Two measures of gastric emptying were obtained by determining *1*) the proportion of rats in which all six beads had exited the stomach for a given treatment over time and *2*) the proportion of the six beads that had exited the stomach.

#### GI transit score.

The rating scale used to classify bead location comprised six beads, each given a numeric score depending on its location within the GI tract: *0*) stomach, *1*) proximal small intestine, *2*) distal small intestine, *3*) cecum, *4*) colon, or *5*) expelled. The total transit score was the sum of the individual bead scores (maximum = 30 if all expelled). The experimenter was blinded to treatment.

#### Transit between regions.

The relative change in GI tract location for each bead between 4 and 9 h and 9 and 12 h was measured according to the difference in location score.

#### Large intestinal transit.

The movement of beads between 9 h (when the majority were in the cecum) and 12 h (when some of the beads had moved to the colon) was observed to assess possible strain differences in colonic transit. The number of beads that had moved from the cecum to the colon over 3 h was determined and compared between strains.

### Sample Collection

Cecal contents were removed and frozen in liquid nitrogen following euthanasia 14 days following the transit measurements to avoid barium contamination. Plasma was prepared from post mortem blood samples, collected by cardiac puncture, by centrifugation at room temperature for 5 min at 1,500 *g* with EDTA used as an anticoagulant. Samples were stored at −80°C until analysis.

### Corticosterone Immunoassay

Plasma corticosterone levels were measured using a commercial kit (Corticosterone ELISA kit; Enzo Life Sciences, Farmingdale, NY) as per the manufacturer’s instructions, and the concentration of each sample was extrapolated from a standard curve. Sensitivity of the assay is <27.0 pg/ml. This was carried out pre- and postacute water stress using the forced swim test (21).

### Microbiota Composition Analysis

Metagenomic DNA was extracted from cecal contents using the NucleoSpin Soil kit (Macherey-Nagel, Düren, Germany) using a previously described method (51). The V3-V4 region of the bacterial 16S rRNA gene was amplified using 16S dual-indexed primers (27). Amplicons were sequenced at NZGL (Palmerston North, New Zealand) using the MiSeq (Illumina, San Diego, CA) with 2× 250-base phosphatidylethanolamine (PE) chemistry. Paired end sequences were joined using the join_paired_ends.py script, and sequences were quality filtered (q30) using the Qiime 1.8 pipeline (10). The resulting sequences were chimera checked using the USEARCH method against the Greengenes alignment (release GG_13_8), following which chimeric sequences were removed from subsequent analyses. Sequences showing 97% or greater similarity were clustered into operational taxonomic units using the UCLUST method, and representative sequences were assigned taxonomies using the Ribosomal Database Project classifier. Differences in mean proportions of taxa were analyzed using nonparametric permutation ANOVA (1,000 permutations per test) as implemented in the RVAideMemoire package (20) in R 3.0.2 (39), the results of which were corrected for multiple testing using the Benjamini and Hochberg false discovery rate (FDR) adjustment. FDR values <0.05 were considered significant.

### Metabolomics Analysis

#### Plasma sample preparation.

The extraction was performed using the method of Amirotti et al. (3), which is capable of generating extracts for both the hydrophilic interaction liquid chromatography (HILIC) and lipid analyses from a single aliquot of plasma. Briefly, 200 µl plasma were extracted by liquid-liquid extraction using a mixture of methanol/chloroform/heptane (2/0.5/0.5 by volume; 600 µl). The sample was vortexed for 30 s, and then, a further 200 µl chloroform and 200 µl water were sequentially added, thoroughly mixing after each addition. The samples were centrifuged for 15 min at 12,500* g* at room temperature to separate the aqueous (upper) and organic (lower) phases. The aqueous phase (200 µl) was taken, dried under a stream of nitrogen and reconstituted in 200 µl of 50:50 acetonitrile:water containing 10 µg/ml d_2_-tyrosine as an internal standard, placed in the LC autosampler at 4°C for HILIC-MS analysis. Likewise, 200 µl of the lower phase were dried under a stream of N_2_ and reconstituted in 200 µl of 2:1 chloroform:methanol (vol/vol) containing a d31-PE internal standard at 10 µg/ml concentration. Blank procedure samples were prepared exactly as the samples, but plasma was replaced with Milli-Q water. To avoid any systematical analytical effects, the samples were randomized before the run. The sequence of runs in each metabolomic analysis comprised blanks, QC (controls), and samples in that particular order. To verify and/or maintain data quality within each mode, a QC sample (comprising a pooled extract of a subset of samples for brain analyses and a bovine plasma sample for plasma analyses) was also injected once for every 10 samples. Retention time, signal/intensity, and mass error of internal standards were monitored constantly to check instrument response variability and retention time shifts.

#### Liquid chromatography-mass spectrometry.

Plasma extracts were analyzed through HILIC and lipid liquid chromatography mass spectrometry (LC-MS) streams using both positive and negative ionization modes. HILIC-MS conditions were as previously described (16). Compounds were separated using a 5 µm ZIC-pHILIC column (100 × 2.1 mm; Merck, Darmstadt, Germany) eluted with *solvent A*: acetonitrile with 0.1% formic acid; and *solvent B*: 16 mM ammonium formate in water, at a flow rate of 250 µl/min. Initial conditions of the solvent gradient were set at 97:3 (*A*:*B*). This was held for 1 min, at which time the gradient was changed linearly to give a ratio of *A* to *B* at 70:30. At 12 min, the gradient was changed linearly to give a ratio of *A* to *B* at 10:90 at 14.5 min, which was maintained for 17 min. At this time and up to 24 min, the column was allowed to reequilibrate and return to the starting conditions. Column effluent was connected to an electrospray source of a high-resolution mass spectrometer (Exactive Orbitrap; Thermo, San Jose, CA), and mass spectral data were collected in profile data acquisition mode covering a mass range of *m*/*z* = 55–1,100 with a mass resolution setting of 25,000 and a maximum trap fill time of 250 ms using the Xcalibur software package (Thermo).

LC-MS conditions were as described by Samuelsson et al. (42). Lipid extracts were separated on an Acquity CSH C_18_ column (100 × 2.1 mm, 1.7-μm inner diameter; Waters, Milford, MA) at 65°C using a gradient elution program at a flow rate of 600 μl/min. The mobile phase consisted of acetonitrile-water-formic acid (59.95:39.95:0.1 vol/vol + 10 mM ammonium formate; *solvent A*) and isopropyl alcohol-acetonitrile-formic acid (99.95:9.95:0.1 vol/vol + 10 mM ammonium formate; *solvent B*) using the following elution program: 85–70% *A* (0–2 min), 70–52% *A* (2–2.5 min), 52–18% *A* (2.5–11 min), 18–1% *A* (11–11.5 min), 1% *A* (11.5–12 min), 1–85% *A* (12–12.1 min), and 85% *A* (12.1–15 min). Column effluent was connected to an electrospray source of a high-resolution mass spectrometer (Q-Exactive Orbitrap; Thermo), and mass spectral data were collected in profile data acquisition mode covering a mass range of *m*/*z* = 200–2,000 with a mass resolution setting of 35,000 and a maximum trap fill time of 250 ms using the Xcalibur software package. A data-dependent MS^2^ (ddMS^2^) fragment analysis using the same settings was performed on every 10th sample to facilitate identification.

#### Data analysis.

Data processing essentially involved a series of procedures aimed at converting raw mass spectrometry data to data matrices suitable for further statistical analyses. This included eliminating background noise, identifying significant peaks, and normalizing data with reference to the internal standard to retain uniformity in the resultant data matrix.

Metabolites eluting between 3 and 18 min for the HILIC analysis and between 1 and 11 min for the lipidomics analysis were extracted from the LC-MS data by converting the data files to mzXML file format (ProteoWizard; ProteoWizard Software Foundation, San Diego, CA) and performing peak detection, alignment, and grouping using XCMS. The resultant peak intensity table was subjected to an in-house linear run-order correction normalization and isotope/adduct annotation, using respective R-based software, and data corresponding to isotopes and background noise, i.e., solvent front and peaks detected in the blanks, were removed from the final data matrix.

#### Statistical analysis.

With the exception of previously described statistical methods, all analyses were carried out using GenStat version 18 (VSN International, Hemel Hempstead, UK) and Minitab 17 Statistical software (Minitab, State College, PA). Results are expressed as means ± SE.

#### Stomach emptying.

Two logistic regression analyses were carried out at each time point (4, 9, and 12 h) using strain as the factor, to compare differences in the proportion of rats with no beads in the stomach (0/1) and the proportion of beads remaining in/exited the stomach.

#### GI transit score.

Data were analyzed at each time point (4, 9, and 12 h) using a linear mixed model (REML) with strain as the factor to compare differences between strains, and Fisher’s least significant differences was used for the post hoc test.

#### Transit between regions.

Data were analyzed using a linear mixed model (REML) with strain and time (9 and 12 h) as factors to compare differences between strains, and Fisher’s least significant differences was used for the post hoc test.

#### Large intestinal transit.

Data were analyzed using ANOVA with strain as the factor, following square root transformation to meet the assumptions of normality and homogeneity.

#### Metabolomics statistical analysis.

Data analysis was performed using a comprehensive online data analysis suite, MetaboAnalyst version 3.0 (McGill Univeristy, Montreal, Quebec, Canada) (50a). Data from the two ionization modes for each of the chromatographic analyses (HILIC and lipid) were combined for statistical analysis. The data were filtered using relative standard deviation filtering, log2-transformed and then autoscaled (mean centered and divided by SD of each variable). Univariate and multivariate data analyses were conducted. Principal component analysis (PCA) was used to get an overview of the two data sets and investigate for possible run order effects. Next, fold change and *t*-test analysis of the strains were performed, and a FDR correction was utilized to reduce the risk of type I errors (false positives). Mass spectral features with FDR <0.1 for HILIC and FDR <0.05 for lipid analysis were considered to differ significantly between strains.

#### Compound identification.

Significant features identified by *t*-tests are presented. Feature annotation for HILIC-MS was performed by matching peaks against a local library of authentic standards run under identical conditions. Where no hit was successful, significant features (as detected by the statistical tests described below) were searched against the public domain databases HMDB and METLIN (a tolerance of 5 ppm was allowed). Lipid LC-MS annotations were performed by matching the XCMS generated data matrix against lipids identified in the samples where ddMS^2^ had been collected, using LipidSearch software (Thermo).

## RESULTS

### Stomach Emptying

#### Sprague-Dawley.

**Fig. 1.**
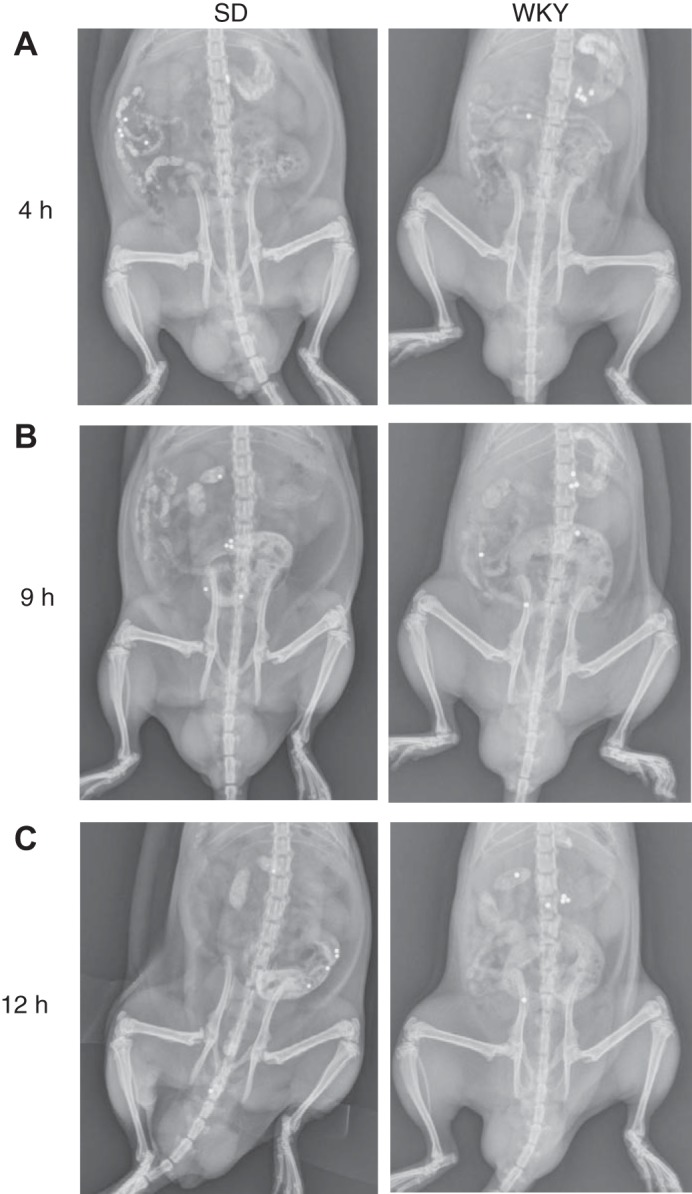
Location of 6 metallic beads over time in Sprague-Dawley (SD) compared with Wistar-Kyoto (WKY) rats. Representative examples of bead locations are shown for X-ray ventral view images at postgavage: 4 h (*A*), 9 h (*B*), and 12 h (*C*).

**Fig. 2.**
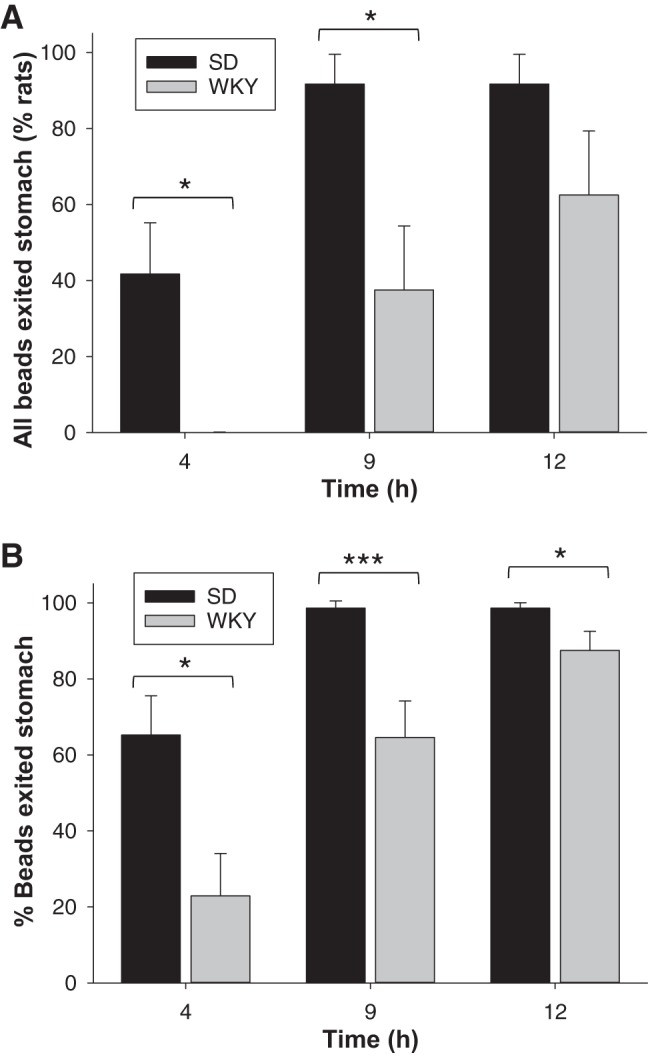
Comparison of transit from the stomach over 12 h for Sprague-Dawley (SD) compared with Wistar-Kyoto (WKY) rats (*n* = 12 animals per group). *A*: percentage of rats in which all beads had exited the stomach. *B*: percentage of beads that had exited the stomach per rat (mean per treatment). Data show means ± SE. *P < 0.05; ***P < 0.01 significance of each treatment relative to controls.

At 4 h postgavage, all beads had exited the stomach in 42% of SD animals, which was calculated as 65% of beads having exited on a per bead basis (Figs. 1*A* and 2). By 9 h, 92% of beads had exited the stomach in 90% of animals (Figs. 1, *B* and *C*, and 2).

#### Wistar-Kyoto.

In contrast, at 4 h postgavage, all WKY animals had at least one bead remaining in their stomach (Figs. 1*A* and 2). The percentage of beads that had exited the stomach at 4 h was only 23% for the WKYs, which was 2.8-fold less than for SD rats (Fig. 2*B*). By 9 h postgavage, WKY animals had 2.9-fold more beads remaining in their stomach than SDs (Fig. 1, *B* and *C*). The stomach location status of the beads over time revealed that in two WKY animals no beads exited the stomach over 12 h, and a two had transit scores of less than three. This subset of animals was excluded from subsequent transit score analysis because no meaningful transit measurements were possible due to stomach emptying being substantially delayed.

### GI Transit

#### Sprague-Dawley.

**Fig. 3.**
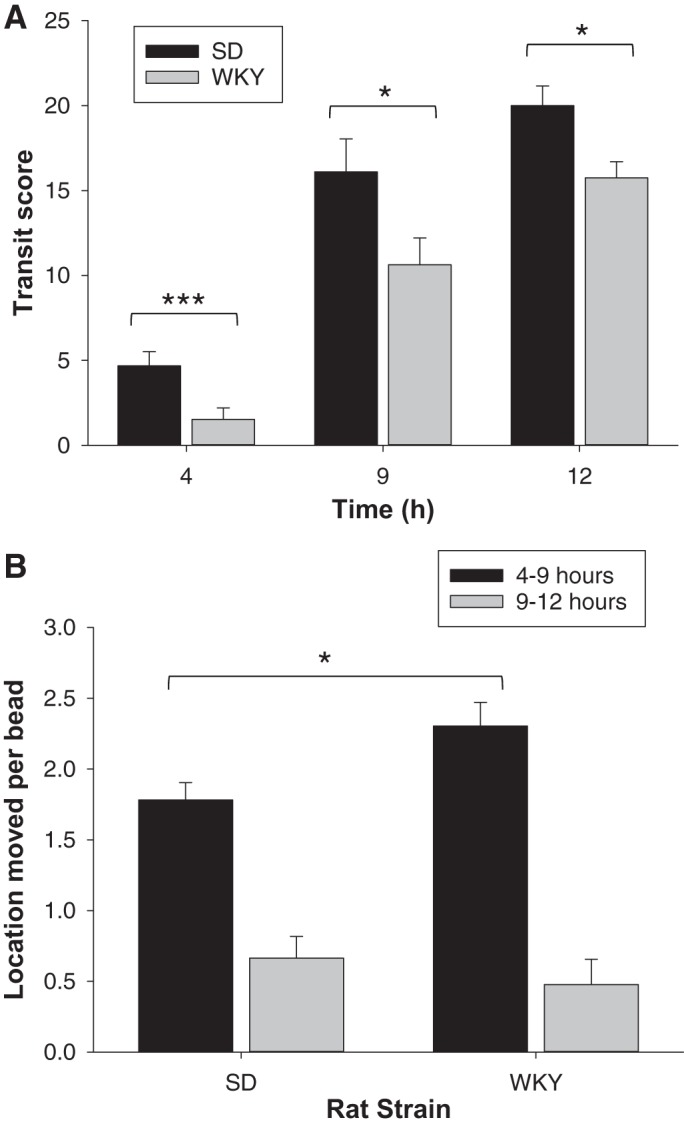
Comparison of gastrointestinal transit tracked over 12 h for Sprague-Dawley (SD) compared with Wistar-Kyoto (WKY) rats (*n* = 8–12 animals per group). *A*: transit scores for 6 solid beads. *B*: location moved per bead. Data show means ± SE. *P < 0.05; ***P < 0.01, significance of each treatment relative to controls.

For SD rats, the bead transit score of 5 at 4 h means that most of the beads were in the proximal small intestine (Figs. 1*A* and 3*A*). The score of 16 at 9 h places approximately half of the beads in the distal small intestine and half in the cecum on average, and the score of 20 at 12 h places them in the large intestine (Figs. 1, *B* and *C*, and 3*A*).

#### Wistar-Kyoto.

Results showed that at 4 h postgavage bead transit along the GI tract of WKY rats was 68% slower, compared with that of SD rats (Figs. 1*A* and 3*A*). At 9 h postgavage, WKY rats had slower GI bead transit than SD rats by 34%, placing the beads in the small intestine (Figs. 1*B* and 3*A*). At 12 h postgavage, WKY had slower GI bead transit than SD rats by 21%, placing approximately half of the beads in the distal small intestine and half in the cecum on average (Figs. 1*C* and 3*A*).

### Bead Movement Between Locations

To determine whether the delayed GI transit for WKY rats was solely a consequence of delayed stomach emptying, the movement of beads between GI tract locations thereafter was assessed. Any beads remaining in the stomach at 9 h were discounted because they did not transit along the GI tract. Results showed that over 4–9 h postgavage beads transited 29% further in WKY than in SD animals, but this was not different at 9–12 h (Fig. 3*B*).

### Cecum to Colon

**Fig. 4.**
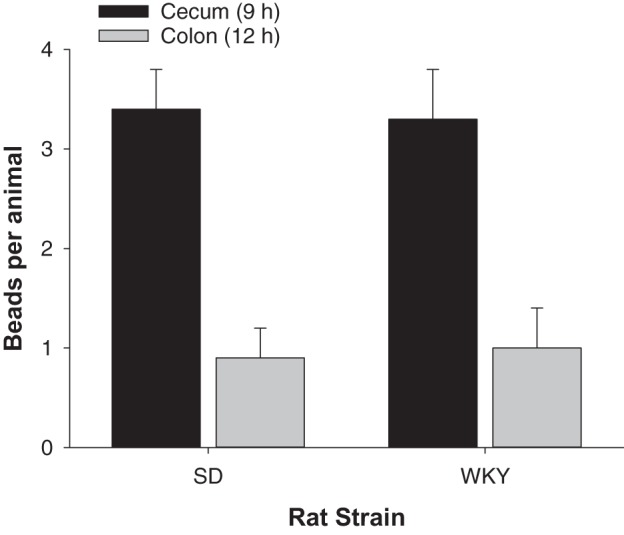
Cecum to colon transit. The number of beads per animal that moved from the cecum at 9 h (black) to the colon at 12 h (gray) are shown for Sprague-Dawley (SD) compared with Wistar-Kyoto (WKY) rats (*n* = 7–11 animals per group). Data show means ± SE.

To measure possible differences in transit to the colon, taking the cecum as a starting point, ~3.5 beads per animal were in the cecum at 9 h in both strains (Fig. 4). By 12 h this was not significantly different between the rat strains in that, on average, one bead had transited per animal from cecum to colon.

### Bead-Size Comparison

**Fig. 5.**
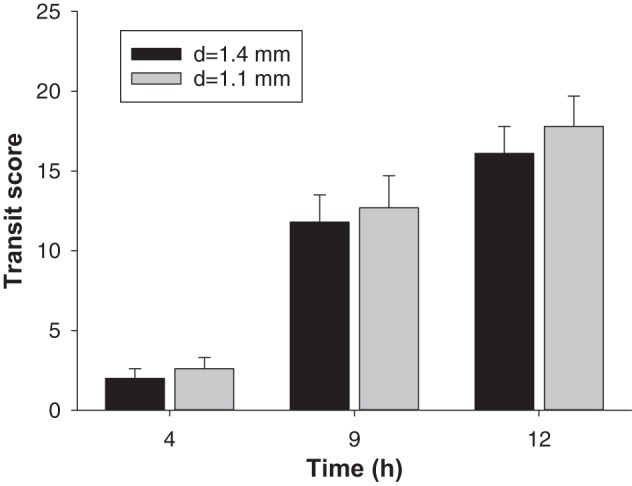
Gastrointestinal transit scores for two different sizes of bead tracked over 12 h in Wistar-Kyoto (WKY) rats (*n* = 12 animals per group). Data show means ± SE; *d*, diameter.

The WKY rats weighed only 264 ± 5 g (*n* = 8) compared with 445 ± 10 g (*n* = 12) for SD rats, and corresponding food intake was 17.7 ± 0.4 g/day (*n* = 8) and 28.2 ± 0.5 g/day (*n* = 12), respectively. Due to the physical size difference between the strains, we explored the possibility that bead retention in the WKY stomach was due to difficulty traversing the pyloric sphincter of this smaller strain. A bead size of 1.1-mm diameter was considered a suitable comparative size because <1-mm diameter has been shown to produce stomach retention problems in rats (22). Transit parameters were assessed for smaller diameter beads (*d* = 1.1 mm) and compared with those for the current size (*d* = 1.4 mm). Results showed that beads were retained in the stomach of all WKY rats at 4 h irrespective of bead size. The proportion of beads that had exited by 4 h was 35 ± 10% (*d* = 1.1 mm) and 22 ± 6% for (*d* = 1.4 mm), which were not significantly different. The other parameters measured, that of transit score (Fig. 5), movement between locations and cecum to colon transit, also did not differ significantly between the two bead sizes.

### Corticosterone Concentrations

No significant difference was found in baseline plasma corticosterone levels between WKY rats (6.9 ± 0.6 ng/ml) and SD rats (6.7 ± 0.8 ng/ml). When the rats underwent acute stress in the form of the forced swim test, plasma levels increased for both WKY rats (18.3 ± 1.3 ng/ml) and SD rats (19.9 ± 1.9 ng/ml). Because the prestress test corticosterone levels were similar between the two rat strains and increased by a similar extent under acute swim stress, the baseline was considered to be a low stress state. Although individual housing cannot be ruled out as a stressor, the low corticosterone levels suggest this effect was minimal.

### Microbiota Composition

**Fig. 6.**
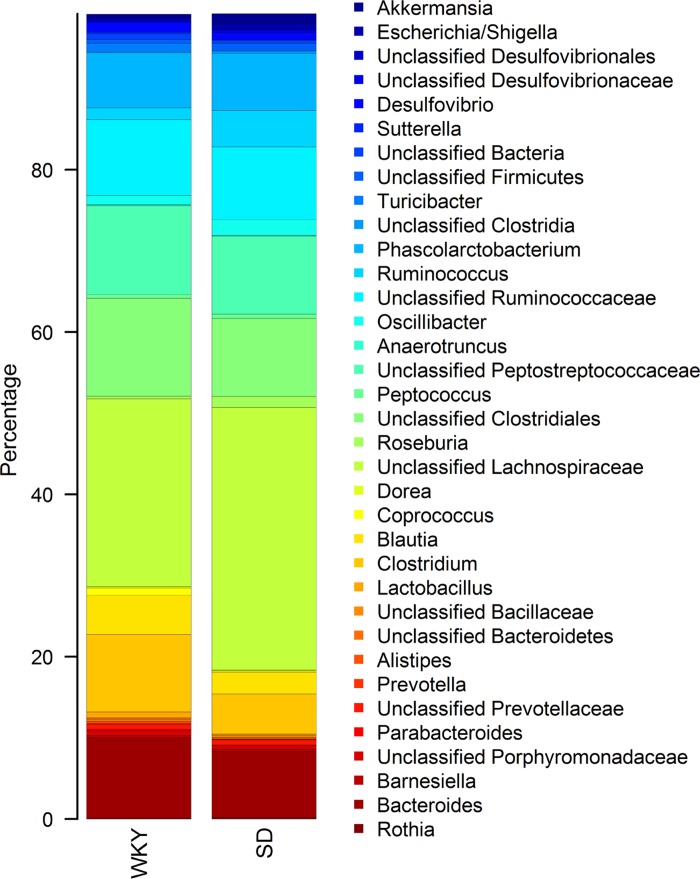
Stacked bar plot of mean relative abundances of genera level taxa that make up >1% of the cecal communities in Wistar-Kyoto (WKY) and Sprague-Dawley (SD) (*n* = 12).

At the phylum level, both SD and WKY rats had a similar cecal microbiota composition typical of that found in rats (18, 52), where *Firmicutes* was the most abundant phylum at 84% for WKY and 86% for SD, followed by *Bacteroidetes* at 13 and 10%, respectively. However, clear differences could be discerned at the family and genus level (Fig. 6 and supplemental data S1; supplemental material for this article is available online at the journal website). Of the taxa that were the most highly represented (>1% mean abundance in either SD or WKY rats), *Ruminococcus*, *Roseburia*, and a group of unclassified *Lachnospiraceae* were significantly (FDR <0.05) less abundant in WKY rats, being 2.8-, 4.1-, and 0.5-fold lower, respectively. Among the minor taxa (<1% mean abundance in either SD or WKY rats), *Dorea* was 2.1-fold higher in WKY compared with SD rats (FDR = 0.02). *Turicibacter* and *Lactobacillus* were 6.6- and 7.7-fold more abundant in WKY compared with SD rats, respectively (FDR = 0.02). Furthermore, *Clostridium* and *Blautia* were 2.0-fold higher in WKY compared with SD rats (*P* < 0.05). Although these two genera were not significantly different after multiple testing adjustments (FDR = 0.08 to 0.09), they made up a substantial proportion of the community, with *Clostridium* accounting for 4.4 and 9.3% in SD and WKY rats, respectively, and *Blautia* making up 2.4 and 4.9% in SD and WKY rats, respectively.

### Plasma Lipid Strain Comparison

**Fig. 7.**
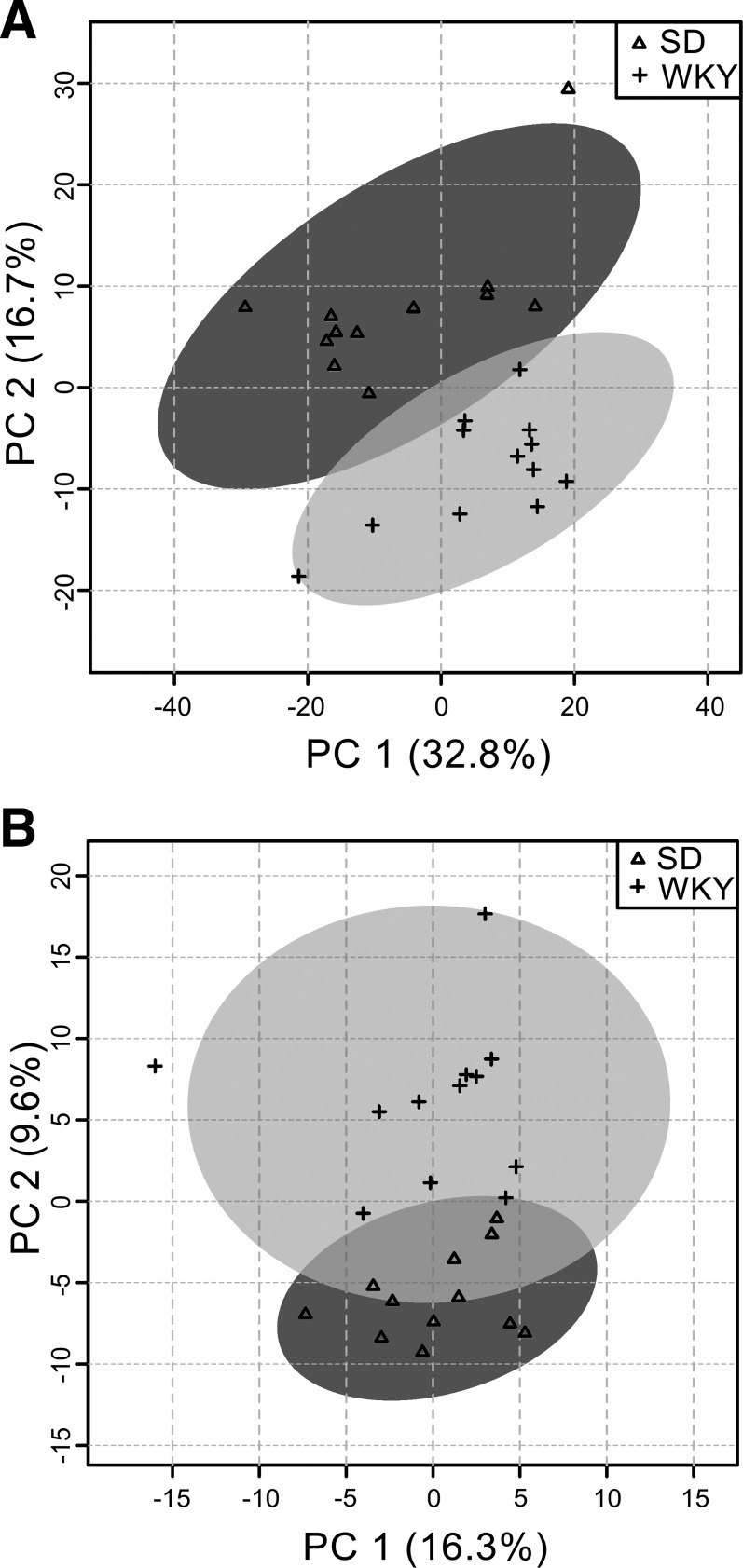
Principal component analysis of combined positive (+ve) and negative (–ve) ionization for plasma lipid species (*A*) and plasma metabolite species (*B*) data. The principal component axis 1 (PC1) and 2 (PC2) show the amount of variation (%) within the metabolomics data explained for each component. Individual data points are shown for Sprague-Dawley (SD) and Wistar-Kyoto (WKY) (*n* = 12).

The combined data matrix of lipid positive and negative mode contained 1,838 unique features for statistical analysis. PCA analysis showed no obvious run-order effects in the data and revealed strong differentiation and clustering of the two strains (Fig. 7*A*). The *t*-tests yielded 277 features with a FDR <0.05 and, of these, 50 were annotated as lipid molecular ion species. In general, diglycerides, triglycerides, PE, and phosphatidylserine were lower in WKY compared with SD rats, while the cholesterol esters, sphingomyelins, sitosterol esters, and two lysophosphocholines (LPC) were higher in WKY compared with SD rats (Table 1 and supplemental data S2).

**Table 1.** Fold change, P value, and FDR for significantly differential plasma lipid species between Wistar-Kyoto and Sprague-Dawley rats

Lipid Class/Lipid ID	*P* Value	FDR	Log2FC (WKY/SD)
Cholesteryl esters			
ChE(16:0) [M+NH4]	0.001389	0.01424	0.35
ChE(18:2) [M+NH4]	0.000134	0.00273	0.49
ChE(18:3) [M+NH4]	9.62E-06	0.00044	0.75
ChE(20:4) [M+NH4]	0.004107	0.03036	0.31
Sitosteryl esters			
SiE(18:2) [M+NH4]_1	0.001831	0.01750	0.77
SiE(18:2) [M+NH4]_2	4.03E-09	0.00000	1.40
SiE(20:4) [M+NH4]_1	0.00208	0.01893	0.35
SiE(20:4) [M+NH4]_2	1.62E-07	0.00001	1.09
Diacylglycerides			
DG(34:1) [M+NH4]	0.000687	0.00860	−1.16
DG(34:2) [M+NH4]	0.007094	0.04728	−0.80
DG(34:3) [M+NH4]	0.001088	0.01169	−0.84
DG(36:2) [M+NH4]	0.00067	0.00850	−0.98
Triacylglycerides			
TG(47:1) [M+NH4]	0.000551	0.00745	−1.05
TG(49:1) [M+NH4]	0.000318	0.00510	−1.24
TG(49:2) [M+NH4]	0.0006	0.00790	−1.10
TG(49:3) [M+NH4]	0.002104	0.01893	−1.00
TG(51:3) [M+NH4]	0.004009	0.02976	−0.81
TG(46:0) [M+NH4]	0.000455	0.00664	−0.99
TG(46:1) [M+NH4]	0.000125	0.00256	−1.89
TG(48:1) [M+NH4]	3.12E-05	0.00104	−1.64
TG(50:4) [M+NH4]	0.001937	0.01823	−0.99
TG(46:2) [M+NH4]	0.000968	0.01084	−1.51
TG(48:0) [M+NH4]	0.000245	0.00406	−1.00
TG(50:1) [M+NH4]	6.89E-05	0.00174	−1.14
TG(48:2) [M+NH4]	9.86E-05	0.00221	−1.76
TG(50:2) [M+NH4]	9.31E-05	0.00214	−0.15
TG(51:2) [M+NH4]	0.000793	0.00934	−1.02
TG(52:2) [M+NH4]	0.000234	0.00391	−0.68
TG(52:3) [M+NH4]	0.001266	0.01344	−0.53
TG(56:7) [M+NH4]	0.003903	0.02940	−0.81
TG(56:8) [M+NH4]	0.001403	0.01424	−0.89
TG(57:2) [M+NH4]	0.00479	0.03445	−0.72
TG(48:3) [M+NH4]	0.000964	0.01084	−1.33
TG(50:3) [M+NH4]	6.96E-05	0.00174	−1.16
TG(53:2) [M+NH4]	0.000534	0.00728	−0.91
TG(53:3) [M+NH4]	0.002436	0.02129	−0.88
TG(50:0) [M+NH4]	0.000582	0.00776	−0.92
TG(52:0) [M+NH4]	0.003696	0.02833	−0.69
TG(52:1) [M+NH4]	0.000216	0.00367	−1.31
TG(54:1) [M+NH4]	0.001736	0.01685	−1.00
TG(54:3) [M+NH4]	0.002776	0.02304	−0.52
TG(59:2) [M+NH4]	0.003913	0.02940	−0.91
TG(58:9) [M+NH4]	0.002046	0.01893	−0.91
Sphingomyelins			
SM(d40:1) [M-HCOO]	0.001744	0.01685	0.61
SM(d41:1) [M-HCOO]	0.003811	0.02900	0.61
Phospholipids			
LPC(20:0) [M+H]	0.006571	0.04395	0.52
LPC(22:5) [M+H]	0.000146	0.00293	0.56
PE(38:6) [M+H]	0.000366	0.00567	−1.24
PS(43:6) [M-H]	0.002714	0.02284	−0.67

Significantly different [false discovery rate (FDR) < 0.05] plasma lipids. WKY, Wister-Kyoto; SD, Sprague-Dawley; Log2FC, log2 fold change.

### Plasma Polar Metabolite Strain Comparison

The combined data matrix of HILIC positive and negative mode contained 1,206 unique features for statistical analysis. PCA analysis showed no obvious run-order effects in the data and revealed strong differentiation and clustering of the two strains (Fig. 7*B*). The *t*-tests yielded 124 features with a FDR <0.1 and, of these, 23 were annotated as molecular ion species. Within the other 101 features not identified, there were ~20 highly correlated source-induced fragment ions to aid with identification. The annotated metabolites along with *P* values, FDR, and fold change are reported in Table 2 (supplemental data S3). Significantly lower levels of 2-methylnicotinamide (a metabolite of vitamin B_3_ metabolism), picolinic acid (tryptophan catabolite), carnitine and its metabolites, seven amino acids (see Table 2), and methylhistidine were observed in WKY relative to SD rats. Other metabolites elevated in WKY rats were B-alanine, dimethylglycine (a by-product of the metabolism of choline, a water-soluble B vitamin), and iminoaspartic acid (a dicarboxylic acid in the biosynthesis of nicotinic acid, vitamin B_3_). Notably, a peak corresponding to the accurate mass and isotope pattern for the taurine-conjugated primary bile acids (taurocholic acid and tauroα/β-muricholic acid), as well as the secondary bile acid tauroω-muricholic acid, was consistently elevated in WKY rats, although this peak was not significant at the FDR <0.1 threshold for Table 2 (*P* = 0.028, FDR = 0.208; supplemental data S3).

**Table 2.** Fold change, P value, and FDR significantly differential plasma metabolite species between WKY and SD rats

Class	Possible ID	*P* Value	FDR	Log2FC (WKY/SD)
Amino acid	B-alanine	2.66E-05	0.000964	1.02
Amino acid	Match: dimethylglycine	1.1E-07	1.66E-05	0.72
Amino acid	4-hydroxy-l-proline	0.006689	0.074007	0.37
Amino acid	Methionine	0.000477	0.009481	−0.26
Amino acid	Match: ornithine	0.005511	0.063902	−0.28
Amino acid	Isoleucine	0.00384	0.049272	−0.29
Amino acid	Match: 1- or 3-methylhistidine	0.004591	0.05674	−0.31
Amino acid	Alanine	0.001559	0.024731	−0.32
Amino acid	Homoserine	0.000284	0.006453	−0.33
Amino acid	Glutamine	0.000368	0.007783	−0.37
Amino acid	l-arginine	0.006946	0.074795	−0.43
Amino acid	Threonine	4.99E-06	0.000287	−0.51
Arginine metabolism	Possibe: 4-guanidinobutanoic acid	2.81E-05	0.000967	−0.75
Carnitine metabolisom	Match: l-acetylcarnitine	0.000903	0.015563	−0.55
Carnitine metabolism	Match: 3-dehydroxycarnitine	0.001228	0.020418	−0.60
Carnitine metabolism	Possible: isobutyryl-l-carnitine	0.002717	0.036821	−0.71
Carnitine metabolism	Match: carnitine	0.001934	0.028448	−0.72
Dicarboxylic acid	Possible: iminoaspartic acid	0.002137	0.030675	0.63
Peptide	Possible: dipeptide Leu-Val	1.5E-07	2.02E-05	1.66
Pyrimidine metabolism	Match: dihydrothymine	0.000771	0.013484	−0.31
Tryptophan metabolism	Match: picolinic acid	1.58E-05	0.000656	−1.28
Vitamin metabolism	Possible: 2-methylnicotinamide	5.09E-09	2.28E-06	−2.25

Significantly different (FDR < 0.1) plasma metabolites.

## DISCUSSION

The main findings of this study are that stomach emptying was delayed in WKY compared with SD rats and that this delayed stomach emptying masked faster small intestinal transit in WKY compared with SD rats. This became evident once beads retained in the stomach were accounted for. Thus slower overall transit was a consequence of delayed stomach emptying. Stomach retention of beads was not due to the larger bead size relative to body weight in WKY rats because comparative experiments ruled this out. No difference was detected in large intestinal transit over 12 h in this study using the cecum as the reference location. This is consistent with a report that there is no difference in fecal output (or number of pellets) between nonstressed WKY and SD rats (21). Our supposition that the WKY rats are in the nonstressed state is supported by the similarly low corticosterone levels detected in both strains.

### Delayed Gastric Emptying

How the delayed gastric emptying detected in WKY rats relates to impaired gastric accommodation previously reported for this strain is unclear (4, 23), although both are features of functional dyspepsia (9). Impaired gastric accommodation has been attributed to increased gastric vagal cholinergic tone (4). It is known that intracerebroventicular administration of corticotropin-releasing hormone (CRF) inhibits gastric emptying in SD rats via the hypothalamic-pituitary-adrenal axis (HPA) (29, 46). Although not specifically addressed in this study, it is known that levels of CRF peptide and corresponding CRF receptor expression are altered in key HPA brain regions in nonstressed WKY rats (compared with SD rats), including the hypothalamus and extrahypothalamic regions such as the hippocampus (7, 35). This dysregulation might contribute to inhibition of gastric emptying via the HPA, most likely through regulation of vagal inhibitory circuits (8). We note that the Flinders-sensitive line rat is used as a model of depression that is hypersensitive to cholinergic stimuli and also exhibits delayed stomach emptying but not altered gastric accommodation (30). Thus delayed stomach emptying and impaired accommodation do not necessarily involve a common mechanism.

Delayed gastric emptying in the absence of a mechanical obstruction is referred to as gastroparesis (37). By using a mix of solid and semisolid gastric contents, the method described here approximates the standard solid phase gastric emptying scintigraphy technique used to measure gastroparesis clinically after meal ingestion (1). A difference is that human measurements are carried out following an overnight fast. This was avoided in the current rat study to maintain active motility and avoid retention of beads in the stomach as has been reported for solid capsules in fasted rats (43); nevertheless, we believe our observations are relevant to the human condition.

Our results suggest that the WKY rat may be a useful model to study gastroparesis. Delayed gastric emptying would be expected to have consequences on the coordinated activity of enteric reflexes and neuronal relays throughout the GI tract that underlie normal digestion (9). The rate of gastric emptying influences dietary lipid absorption; thus when gastric emptying is delayed absorption may occur more slowly. Because gastroparesis is considered a complex, multifactorial, chronic, digestive disease state, multiple physiological alterations can occur. The metabolomic and microbiota consequences of gastroparesis have not been reported in humans; thus differences in an animal model might provide insights to this condition.

### Microbiota Comparison

The main differences we found in the cecal communities between SD and WKY rats appear to involve taxa known to be involved in the degradation of starch or other plant-based polysaccharides (15, 48, 53), producing short-chain fatty acids as a fermentation by-product. The *Lachnospiraceae* and *Ruminococcaceae* are particularly abundant members of the large intestine microbiota in humans and rodents that play a key role in polysaccharide degradation (15, 47, 50) and include butyrate-producing bacteria such as *Roseburia* (2), which in our study was decreased in WKY rats. These variations in microbiota composition did not correlate with colonic transit since this did not differ between rat strains.

*Turicibacter*, a member of the *Erysipelotrichaceae* family, which was higher in WKY, rats has been associated with high-fat diets (14, 28). *Lachnospiraceae* have also been reported to be increased in response to high-fat diets (13). Other taxa that have been shown to respond to high-fat diets include *Blautia*, *Ruminococcus*, and *Dorea* (54), and all differed between WKY and SD rats in our study. In addition to its well-known role for producing lactic acid from fermentation of carbohydrates, *Lactobacillus*, which was increased in WKY relative to SD rats, is also known to express bile salt hydrolases. Bacterial bile salt hydrolase activity can influence lipid metabolism, weight gain, and cholesterol levels through a variety of mechanisms including altering host signaling pathways and bile salt recirculation and increasing de novo synthesis of bile salts produced from cholesterol (6, 26, 40). Our results suggest that differences in the microbiota between SD and WKY rats may have a functional significance for lipid metabolism and response to dietary fat.

The possibility of altered bile acid metabolism by the WKY microbiota is relevant for gastric emptying because increased levels of circulatory bile acids (usually induced by high-fat diets) upregulate nitric oxide synthase and bile acid receptor 1 (TGR5) expression in the gastric myenteric plexus, resulting in enhanced nonadrenergic noncholinergic relaxation and delayed gastric emptying in rats (55). We propose that the increased *Lactobacillus* in WKY microbiome can contribute to delayed gastric emptying and disregulation of lipid metabolism via bile salt signaling, although further studies are required to clarify this.

### Plasma Metabolite and Lipid Comparison

The higher plasma cholesterol in WKY compared with SD rats further suggests differences in lipid metabolism status between these strains. While strain differences have not been reported previously specifically for lipid metabolism, higher liver hepatic lipase levels and lower adrenal hepatic lipase levels have been reported for WKY compared with SD rats (17). Higher plasma cholesterol in WKY rats could either be due to higher absorption in the small intestine or higher endogenous production in the liver and gall bladder (25). Taken together, our plasma lipid and metabolite results suggest that bile acid pool regulation is impaired in WKY rats.

Increased plasma taurocholic acid in WKY compared with SD rats, together with the altered microbiota findings, suggests a disruption to the bile acid-gut microbiome axis. Lower levels of 2-methylnicotinamide, a metabolite of vitamin B_3_ important for fat metabolism, in WKY rats may suggest alterations in fatty acid and cholesterol synthesis pathways. Consistent with this are the lower levels of di- and triglycerides and PEs but higher cholesterol esters, sphingomyelins, and sitosterol esters, in WKY compared with SD rats. Lower plasma picolinic acid levels in WKY compared with SD rats are consistent with alterations in bile acid secretion into the small intestine. The physiological relevance of the other plasma metabolite differences between strains is less obvious, although we note that l-acetylcarnitine is involved in phospholipid metabolism and modulation of neurotransmission and is thought to be beneficial in depressive disorders (38).

A contributing factor to the increased host cholesterol synthesis in WKY compared with SD rats might be an alteration in the microbiota that increases plasma cholesterol through an increase in bile salt formation. In the rat, feedback to decrease gastric emptying is mediated by a vagal afferent pathway. Chylomicron formation is important in the signaling of lipids in the intestinal lumen to CCK endocrine cells and producing the feedback inhibition of gastric emptying. Our findings suggest that in the WKY rat the negative feedback signaling pathways that normally regulate gastric emptying are overactive. This supports the idea that the measured changes in GI transit and plasma lipids and metabolites reflect dysbiosis of lipid metabolism anticipated to result in elevated bile acids, high plasma cholesterol, and increased chylomicron levels, contributing to inhibition of gastric emptying. It is not clear from this study which factors are causative in this process. To elucidate rat strain differences in the gut-microbiome axis, we would need to also examine primary bile acid production in the liver and compare plasma and fecal secondary bile pools to assess absorption and microbial bile acid modification. Furthermore, because TGR5 (G-protein coupled receptor specific for bile acids) has a higher affinity for secondary compared with primary bile acids (5, 12), secondary bile acids are more likely to affect gastric emptying and GI motility and will be the focus of future studies.

### Conclusion

Our comprehensive approach combining GI physiology, microbial genomics, and metabolomics has provided new insights into the WKY phenotype as characterized by gastroparesis. The associated lipid metabolic dysbiosis implicates bile acid disregulation of fatty acid metabolism and cholesterol turnover. Our findings demonstrate impaired gastric emptying, yet rapid small intestinal transit, as hallmarks of WKY rat physiology under basal conditions. These strain differences in GI transit, microbiota, and plasma lipids suggest that WKY rats may be an appropriate model for gastroparesis and suggest that bile acid metabolism warrants further investigation.

## GRANTS

This study was funded by Strategic Science Investment Fund (Contract No. A21246) from AgResearch, Palmerston North, New Zealand.

## DISCLOSURES

No conflicts of interest, financial or otherwise, are declared by the authors.

## AUTHOR CONTRIBUTIONS

J.E.D., K.F., W.Y., S.A.B., and N.C.R. conceived and designed research; J.E.D., K.F., and W.Y. performed experiments; J.E.D., K.F., W.Y., and C.M.M. analyzed data; J.E.D., K.F., and W.Y. interpreted results of experiments; J.E.D., K.F., and W.Y. prepared figures; J.E.D. drafted manuscript; J.E.D., K.F., W.Y., C.M.M., S.A.B., and N.C.R. edited and revised manuscript; J.E.D., K.F., W.Y., S.A.B., and N.C.R. approved final version of manuscript.

## Supplementary Material

Supplemental_Data_1.xlsx

Supplemental_Data_2.xlsx

Supplemental_Data_3.xlsx
